# Crizotinib sensitizes the erlotinib resistant HCC827GR5 cell line by influencing lysosomal function

**DOI:** 10.1002/jcp.29463

**Published:** 2020-01-20

**Authors:** Nele Van Der Steen, Kaylee Keller, Henk Dekker, Letizia Porcelli, Richard J. Honeywell, Johan Van Meerloo, René J. P. Musters, Ietje Kathmann, Adam E. Frampton, Daniel S. K. Liu, Rob Ruijtenbeek, Christian Rolfo, Patrick Pauwels, Elisa Giovannetti, Godefridus J. Peters

**Affiliations:** ^1^ Center for Oncological Research University of Antwerp Antwerp Belgium; ^2^ Department of Pathology Antwerp University Hospital Antwerp Belgium; ^3^ Laboratory of Medical Oncology Amsterdam Universities Medical Centers, VUmc Amsterdam The Netherlands; ^4^ Experimental Pharmacology Laboratory IRCCS Istituto Tumori "Giovanni Paolo II" Bari Italy; ^5^ Department of Pediatric Oncology/Hematology VUmc Amsterdam The Netherlands; ^6^ Amsterdam University Medical Centers, VUmc Amsterdam The Netherlands; ^7^ Division of Cancer, Department of Surgery & Cancer Imperial College London United Kingdom; ^8^ Department of Clinical & Experimental Medicine, Faculty of Health & Medical Sciences University of Surrey Guildford United Kingdom; ^9^ Pamgene International BV PamGene ‘s‐Hertogenbosch The Netherlands; ^10^ Phase I‐Early Clinical Trials Unit, Oncology Department Antwerp University Hospital Antwerp Belgium; ^11^ Cancer Pharmacology Lab, AIRC Start‐Up Unit Fondazione Pisana per la Scienza Pisa Italy

**Keywords:** cMET, crizotinib, EGFR, erlotinib, lysosomes, tyrosine kinase inhibitors

## Abstract

In non‐small cell lung cancer, sensitizing mutations in epidermal growth factor receptor (EGFR) or cMET amplification serve as good biomarkers for targeted therapies against EGFR or cMET, respectively. Here we aimed to determine how this different genetic background would affect the interaction between the EGFR‐inhibitor erlotinib and the cMET‐inhibitor crizotinib. To unravel the mechanism of synergy we investigated the effect of the drugs on various parameters, including cell cycle arrest, migration, protein phosphorylation, kinase activity, the expression of drug efflux pumps, intracellular drug concentrations, and live‐cell microscopy. We observed additive effects in EBC‐1, H1975, and HCC827, and a strong synergism in the HCC827GR5 cell line. This cell line is a clone of the HCC827 cells that harbor an EGFR exon 19 deletion and has been made resistant to the EGFR‐inhibitor gefitinib, resulting in cMET amplification. Remarkably, the intracellular concentration of crizotinib was significantly higher in HCC827GR5 compared to the parental HCC827 cell line. Furthermore, live‐cell microscopy with a pH‐sensitive probe showed a differential reaction of the pH in the cytoplasm and the lysosomes after drug treatment in the HCC827GR5 in comparison with the HCC827 cells. This change in pH could influence the process of lysosomal sequestration of drugs. These results led us to the conclusion that lysosomal sequestration is involved in the strong synergistic reaction of the HCC827GR5 cell line to crizotinib–erlotinib combination. This finding warrants future clinical studies to evaluate whether genetic background and lysosomal sequestration could guide tailored therapeutic interventions.

## Graphical abstract

In non‐small cell lung cancer, sensitizing mutations in epidermal growth factor receptor (EGFR) or cMET amplification serve as good biomarkers for targeted therapies against EGFR or cMET, respectively. Here we aimed to determine how this different genetic background would affect the interaction between the EGFR‐inhibitor erlotinib and the cMET‐inhibitor crizotinib. Both genetic characteristics and lysosomal function seem to contribute to the synergistic interaction between crizotinib and erlotinib in the HCC827GR5 cell line, and more research is warranted to determine the exact mechanisms linking these cellular properties.

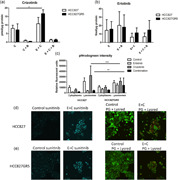

## 1 INTRODUCTION

Non‐small cell lung cancer (NSCLC) consists of three histological subtypes: adenocarcinoma, squamous cell carcinoma, and large cell carcinoma, and has a very poor 5‐year survival rate of around 10% (Travis, Brambilla, Burke, Marx, & Nicholson, 2015). Targeted therapies with tyrosine kinase inhibitors (TKI) have recently revolutionized the treatment of the adenocarcinoma subtype (Hirsch, Suda, Wiens, & Bunn, 2016). Sensitizing mutations (L858R, exon 19 deletions) in the epidermal growth factor receptor (EGFR) are good predictors for response to EGFR‐TKIs such as erlotinib, gefitinib, and afatinib (Pao et al., 2004).

However, individual resistance to these targeted therapies occurs on average within a year, with a mechanism involving acquired mutations, such as the common EGFR‐T790M mutation. The third‐generation EGFR‐TKI osimertinib has been approved for the treatment of T790M positive NSCLC (Jänne et al., 2015) and is recently approved as first‐line treatment (Passiglia, Raez, & Rolfo, 2018). Amplification of cMET is another genetic aberration that leads to resistance to EGFR‐TKIs (Engelman et al., 2007; Ou, Agarwal, & Ali, 2016). Besides functioning as a resistance mechanism, cMET is also known as an oncogenic driver itself. Hereby, amplification of the cMET gene or exon 14 skipping serve as good biomarkers for cMET‐TKIs (Camidge et al., 2014; Lutterbach et al., 2007; Van Der Steen et al., 2016). The dual ALK‐cMET inhibitor crizotinib has been approved for the treatment of ALK‐rearranged NSCLC and is currently undergoing trials as a treatment for cMET‐driven NSCLC (Bahcall et al., 2016).

The physicochemical properties of a drug can influence its activity. This is the case for hydrophobic drugs with a pKa of around 9 (Da Silva, Honeywell, Dekker, & Peters, 2015; De Klerk, Honeywell, Jansen, & Peters, 2018). Most of these drugs can diffuse freely through the plasma membrane and the membranes of intracellular compartments, but several ATP binding cassette (ABC) transporters, such as P‐glycoprotein (PgP) and breast cancer resistance protein (BCRP) may mediate efflux from the cytosolic compartment into cellular organelles such as lysosomes (De Klerk et al., 2018). Since the lysosomes have a very acidic environment, with a pH of around 5, these drugs are protonated and cation‐trapping takes place, also known as lysosomal sequestration (Zhitomirsky & Assaraf, 2015b). The acidity of the lysosomes is caused by the activation of the vacuolar‐type H^+^‐ATPase. In model systems, this proton pump can be inhibited by bafilomycin A1. If the intracellular drug concentrations decrease significantly after the addition of bafilomycin A1, then lysosomal sequestration is involved (Da Silva et al., 2015; Honeywell et al., 2010). Since most of the targets of TKIs are situated at the plasma membrane, lysosomal sequestration prevents their inhibitory role (Zhitomirsky & Assaraf, 2016).

ABC transporters, such as PgP, multidrug resistance‐associated proteins (MRPs) and BCRP can also mediate resistance by effluxing drugs out of the cells. These efflux pumps are located on the cellular membranes, have high expression in gut epithelium and their primary role is to remove xenobiotics from the body (Sharom, 2008). However, many tyrosine kinase inhibitors are also substrates for transport by PgP (Da Silva et al., 2015; De Klerk et al., 2018). There is a clear need to clarify the role of these pumps in limiting the efficacy of these therapies, as well as to generate new strategies, such as lysosomal photo‐destruction of MDF cells overexpressing PgP (Nowak‐Sliwinska et al., 2015).

Here, we investigated the combined effect of erlotinib and crizotinib on various cell lines with different properties and aimed to unravel new mechanisms of synergy in the HCC827GR5 cells. The parental HCC827 cell line is a NSCLC adenocarcinoma cell line harboring an EGFR exon 19 deletion, which is a sensitizing mutation of EGFR. This cell line has been made resistant to gefitinib through prolonged exposure with increasing dosages, which resulted in cMET amplification (Engelman et al., 2007), and combined treatment with the EGFR inhibitor gefitinib and the cMET inhibitor PHA‐665752, which resulted in substantial growth inhibition (Engelman et al., 2007). In the present study, we have shown a strong synergism with two compounds commonly used in the clinical setting (erlotinib and crizotinib), and determined, for the first time, the potential role of lysosomes in this pharmacological interaction.

## 2 MATERIALS AND METHODS

### 2.1 Cell lines and drugs

The HCC827 and H1975 cell lines were purchased from ATCC. The EBC‐1 cell line was purchased from JCRB. The HCC827GR5 cell line was a kind gift of Dr. Pasi A. Jänne (Dana‐Farber Cancer Institute, Boston, MA). Cell line properties, including cMET and EGFR status, are summarized in Table 1.

**Table 1:** Cell line properties

	H1975	EBC‐1	HCC827GR5	HCC827
Histology	Adeno	Squamous	Adeno	Adeno
cMET	Wild‐type	Amplified	Amplified	Wild‐type
Crizotinib IC_50_	4 ± 0.1 µM	50 ± 2 nM	>5 µM	>5 µM
EGFR	L858R + T790M	Wild‐type	Exon 19 del	Exon 19 del
Erlotinib IC_50_	>10 µM	>10 µM	6.6 ± 1.6 µM	40 ± 20 nM

*Note*: To determine the sensitivity to crizotinib and erlotinib cells were treated during 72 hr with several concentrations of crizotinib (0–5 µM) or erlotinib (0–10 µM). Curves were plotted in GraphPad prism v5 and IC_50_ dosages were determined.

Cell lines were maintained in culture flasks (Greiner Bio‐One GmbH, Frickenhausen, Germany) at 37°C in an atmosphere of 5% CO_2_. The EBC‐1 cell line was cultured in DMEM supplemented with 10% fetal bovine serum (BioWest, Nuaillé, France), 100 IU/ml penicillin and 100 µg/ml streptomycin. The HCC827, HCC827GR, and H1975 were cultured in RPMI1640 with 10% fetal bovine serum, 20 mM HEPES and 100 IU/ml penicillin and 100 µg/ml streptomycin.

Crizotinib and erlotinib were purchased from SelleckChem (Houston, TX). Drugs were reconstituted in dimethyl sulfoxide (DMSO), and diluted in phosphate buffered saline (PBS) immediately before use. Bafilomycin A1 was purchased from LC‐Laboratories (Woburn, MA).

### 2.2 Sulforhodamine B assay (SRB) and combination index

The sulforhodamine B assay was used to determine growth inhibition after mono‐ and combination therapy (Bijnsdorp, Giovannetti, & Peters, 2011; Sciarrillo et al., 2016). Cells were plated in a 96‐well plate (Greiner Bio‐One GmbH), using 2000–5000 cells per well. Cells were treated the next day with either 0.1% DMSO as control, erlotinib (0–10 µM), crizotinib (0–5 µM) or their combination. To evaluate if the pharmacological inhibition of MRP1 might affect the sensitivity to crizotinib in HCC827 cells, we performed further experiments with the MRP1 inhibitor MK571, at 20 µM, as described previously (Assaraf et al., 2003). All cells were treated for 72 hr and all experiments were repeated at least three times.

After the determination of the IC_50_ values for erlotinib and crizotinib monotherapy, cells were treated with fixed concentrations (IC_20_ and IC_40_) of crizotinib in combination with a range of erlotinib (0–10 µM). The combination index (CI) was calculated with Calcusyn (Biosoft, Cambridge, UK), based on the method of Chou and Talalay as described previously (Bijnsdorp et al., 2011). This method takes into account the curves of the monotherapies at the respective concentrations to determine the effect of the combination. Fraction affected (FA) was determined at each dose and only values with an FA ≥ 0.5 were used to calculate an average combination index (CI). A CI < 0.8 represents a synergistic combination, 0.8 < CI < 1.2 is additive and CI > 1.2 is antagonistic.

### 2.3 Wound healing assay

Cells were seeded in a confluent layer in 96‐well plates and allowed to attach. The scratch was made using the sterile scratch tool (Peira Scientific Instruments, Belgium). Detached cells were washed away and new medium was added to the wells. Plates were photographed with the Acumen eX3 laser scanner imaging cytometer (TTP‐LabTech Lts, UK). Cells were treated immediately after imaging with 10 µM erlotinib, 5 µM crizotinib or their combination. Plates were incubated at 37°C and periodically photographed to monitor the scratch area. The experiment was terminated after 8 hr for HCC827 and 16 hr for HCC827GR5 cells, due to gap closure. The scratch area was analyzed with the ImageJ software (ImageJ, U.S. National Institutes of Health, Bethesda, MD; https://imagej.nih.gov/ij/, 1997–2018.NIH; Schindelin et al., 2012).

### 2.4 Cell cycle analysis

Cells were seeded in a six‐well plate and treated with drugs as previously described. After 24 and 48 hr cells were collected in round‐bottom FALCON tubes (BD, Franklin Lakes, NJ) and redissolved in 0.5 ml of a propidium iodide solution (50 µg/ml PI, 0.1% sodium citrate, 0.1% Triton X‐100 and 0.1 mg/ml ribonuclease‐A). The analysis was performed on a BD FACS calibur and data were analyzed with CELLQuest™‐Pro software.

### 2.5 Spheroid assay

Flat‐bottom 96‐well plates were coated with sterile unsupplemented heated medium containing 1.5% agarose and allowed to dry for 20 min. Cells were seeded at 5–20 × 10^4^ cells/well depending on the optimal spheroid size and given 3 days to form three‐dimensional structures, that we named spheroids. Next, these spheroids were treated with 5 µM crizotinib, 10 µM erlotinib or their combination. To investigate the effects of these drugs on cells organized in the spheroids, we evaluated the amount of light passing through, as recently described (Sciarrillo et al., 2019). Images of spheroids were taken with an automated phase‐contrast microscope (Acumen eX3 laser‐scanner). Pixel intensities of 8‐bit black/white‐converted images were calculated using the ImageJ software and expressed as mean gray value (i.e., the sum of all gray values of the spheroid selection divided by the pixels of that selection). The “cell aggregation” for each drug‐treated spheroid was then calculated by normalizing for the mean gray values of the sum control spheroids.

### 2.6 Pathscan intracellular signaling array (fluorescent read‐out)

The PathScan sandwich enzyme linked immunosorbent assay (ELISA) was purchased from Cell Signaling Technology (Leiden, The Netherlands) and used according to manufacturer's instructions. Cells were seeded and treated for 24 hr and lysed in lysis buffer with 1 mM phenylmethylsulfonyl fluoride (PMSF). The glass slide was blocked and the lysate was added to the wells. The slide was washed thoroughly and incubated with the detection antibody cocktail. The fluorescent signal was determined using the LI‐COR Biosciences Odyssey imaging system. The ArrayVision software was used to determine pixel intensity.

### 2.7 Peptide tyrosine kinase activity array

The kinase activity of 144 kinases was determined with the PamGene tyrosine kinase activity array. Cells were seeded in 25 cm^2^ flasks and treated with erlotinib, crizotinib or their combination for 24 hr. Cells were lysed and scraped in M‐PER buffer containing phosphatase and protease inhibitors (Thermo Scientific, Rockford, IL). Samples were centrifuged and the supernatant was stored at −80°C. Control sample mix was prepared using ABL buffer (Westburg), 100 µM adenosine triphosphate (Sigma‐Aldrich), and fluorescein‐labeled antibody PY20 (Exalpha, Maynard, MA). Five µg of lysate protein was used for the analysis. The arrays were blocked with 2% BSA before sample loading. The activity was measured at 30°C over 60 cycles using a Pamstation12 (PamGene, ‘s‐Hertogenbosch, The Netherlands). Imaging was performed after each cycle with a 12‐bit charge‐coupled device (CCD) camera so as to monitor the fluorescence intensities in real‐time. Intensities were fit to calculate the final phosphorylation by Bionavigator software version 6.1.

### 2.8 Intracellular drug concentration determination

The intracellular drug concentration was measured as previously described (Honeywell et al., 2010). Cells were treated with the drugs for 24 hr. Pellets were thawed by adding 160 µl of cold phosphate buffer and homogenized. Samples were vortexed and 100 µl was mixed with 400 µl acetonitrile. Samples were incubated for 20 min on ice, vortexed, and centrifuged at 21,000*g* at 4°C for 10 min. Next, 100 µl of the sample was transferred to a 96‐well plate for LC injection and analyzed (Honeywell et al., 2010).

### 2.9 Western blot analysis

Cells were seeded and treated with the drugs for 24 hr. Cells were lysed in lysis buffer (Cell Signaling Technology) supplemented with 1 mM PMSF on ice for 5 min. Next, cells were dislodged using a cell scraper, lysates were sonicated three times for 10 s and spun down for 10 min at 4°C, 14,000*g*. Supernatants were transferred and either used immediately or stored at −80°C.

Samples were separated by sodium dodecyl sulfate polyacrylamide gel electrophoresis at 100 V for 1 hr using a TGX‐precast gel (BioRad, Veenendaal, The Netherlands). Wet transfer to a PVDF membrane was performed at 200 mA for 2 hr.

The experiments were performed with the following antibodies: anti‐p‐cMET (Tyr1234/1235), rabbit, 1:1000 (clone D25); anti‐p‐EGFR (Tyr1068), mouse, 1:1000 (clone 1H12); anti‐mouse‐HRP and anti‐rabbit‐HRP, 1:2000 (Cell Signaling Technologies); JSB1, mouse, 1:500; MRP‐R1, rat, 1:500; anti‐rat‐HRP were kind gifts from Dr. G. Scheffer (Scheffer et al., 2000). For the analysis of MRP1 we included a positive control, as described previously (Lemos et al., 2008).

### 2.10 Live cell fluorescence microscopy

Cells were seeded in Lab‐Tek II Chambered coverglasses grade 1.5 (Thermo Scientific, Rockford, IL) and allowed to attach overnight. Cells were treated for 24 hr with 10 µM erlotinib, 5 µM crizotinib or their combination. The next day, the cells were washed with PBS and indicator free IMDM medium was added. Staining was performed with 5 µM sunitinib (LC Laboratories, Woburn, MA), 0.5 µM Lysotracker Red (Thermo Scientific), and pHrodoGreen (LifeTechnologies). In a first step sunitinib and/or lysotracker red were added in cell medium without indicator and cells were incubated at 37°C for 30 min. pHrodoGreen was added in the second phase and cells were incubated again for 30 min at 37°C. Next, the medium was removed and cells were washed three times with PBS after which a new medium without indicator was added to the cells and imaging was performed on a Leica TCS SP8 STED 3X microscope. Each sample was divided into a number of focus planes: a z‐stack. Z‐stacks were imaged at brightfield, 445 nm (laser power 2) for sunitinib, 488 nm (laser power 0.7) for pHrodoGreen and 561 nm (laser power 1.5) for lysotracker Red.

**Figure 1:**
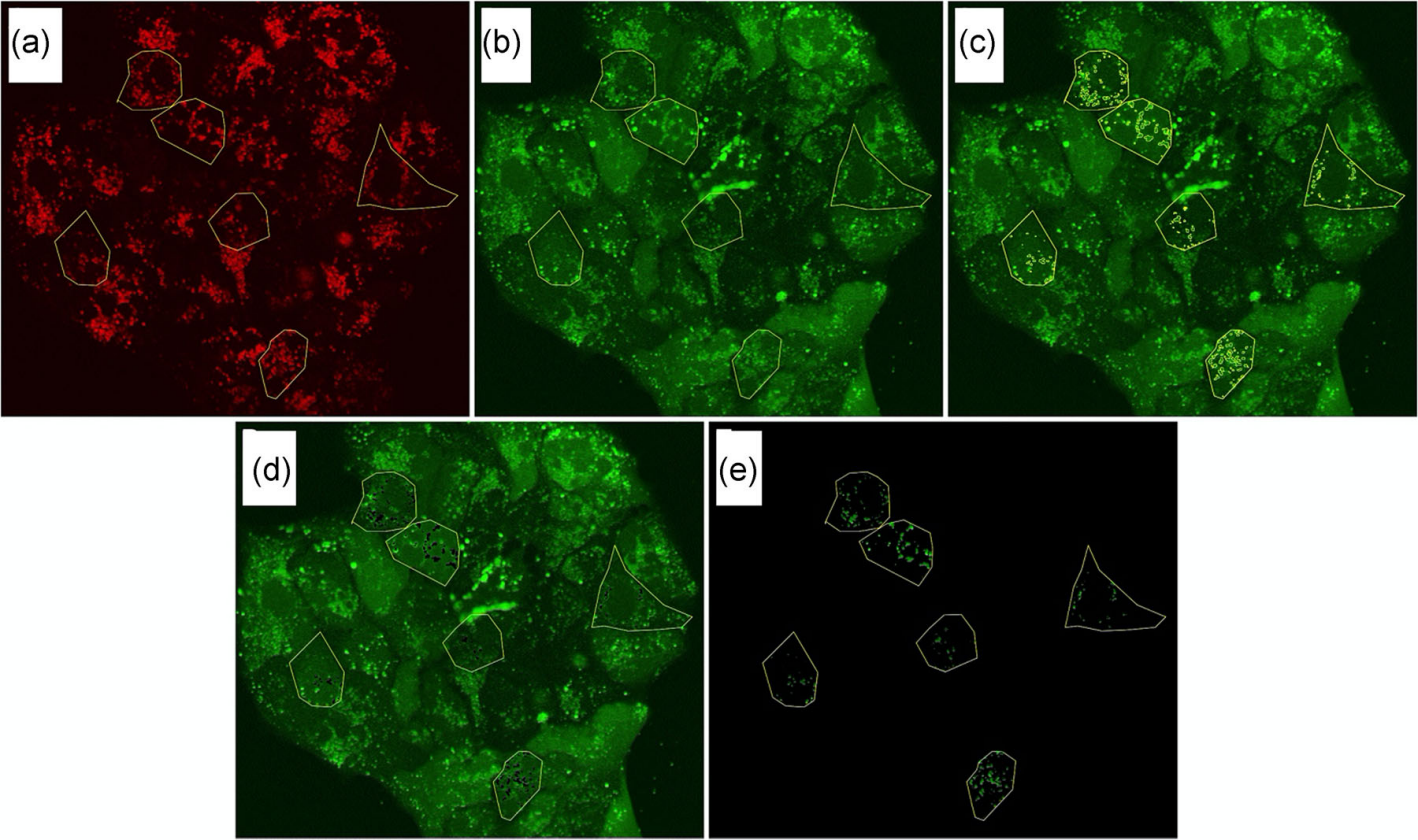
The methodology of FIJI analysis: Fluorescent images were taken of live cells simultaneously treated with Lysotracker Red, pHrodogreen and sunitinib to image pH‐differences and drug uptake. Here we provide guiding images to clarify our analytical method. Images of the Lysotracker Red and pHrodoGreen channel are shown. Yellow markings represent the regions of interest. (a) Lysotracker Red with yellow markings depicting six selected cells for analysis; (b) pHrodoGreen image with yellow markings for six selected cells; (c) pHrodoGreen with markings for six selected cells with markings for lysosomes; (d) pHrodoGreen image with six selected cells with deleted lysosomal intensity; (e) pHrodoGreen image of lysosomes of the six selected cells

FIJI software was used for image analysis (Schindelin et al., 2012; Schindelin, Rueden, Hiner, & Eliceiri, 2015), importing z‐stacks with Bio‐Formats. Ten representative cells were selected per sample (Figure 1a,b). In each z‐plane, the lysosomes were located in the Lysotracker Red channel (Figure 1a), which was converted to binary and signals were automatically traced by the “analyze particle” tool with the threshold “triangle.” The selected regions were overlayed with the pHrodoGreen image (Figure 1b,c). First, the selected regions were deleted from the pHrodoGreen image and the remaining intensity was determined (Figure 1d). Secondly, the outer regions were deleted and the intensity of the lysosomes was measured (Figure 1e). These analyses were repeated for each of the Z‐planes per sample. The intensities were summed and corrected for the area of the cells/lysosomes and the number of z‐planes.

### 2.11 Statistics

Statistical analysis was performed with Graphpad Prism v5 using a one‐way analysis of variance with Tukey's post hoc testing, with *p* < .05 considered as significant. All tests were performed in triplicate unless mentioned otherwise. Values represent mean ± standard error of mean.

## 3 RESULTS

### 3.1 Sensitivity and drug interaction

**Figure 2:**
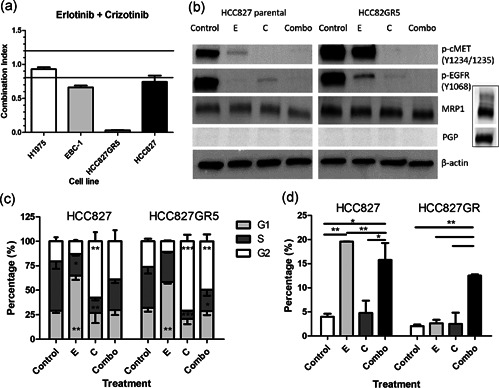
Effect of the erlotinib–crizotinib combination. This figure combines the results of different experiments, evaluating the effect of erlotinib, crizotinib or the combination of growth inhibition, protein phosphorylation, cell cycle arrest, and apoptosis. (a) Combination indexes (CIs) for erlotinib plus crizotinib after 72 hr of treatment. The upper line represents an antagonistic CI > 1.2, the lower bar represents a synergistic CI < 0.8. (b) Protein expression as determined by western blot analysis after treatment with erlotinib and crizotinib (pictures result from the same blot). The insert shows an included positive control To exclude that the lack of signal was due to lack of expression or an issue with the western blot analysis itself. (c) Cell cycle distribution was measured by flow cytometry after propidium iodide staining. (d) Apoptosis after 24 hr treatment, as assessed by the analysis of the sub‐G1 fraction. Control: 0.1% dimethyl sulfoxide, E: 10 µM erlotinib, C: 5 µM crizotinib, Combo: 10 µM erlotinib + 5 µM crizotinib. *p < .05 as compared to control, **p < .01, ***p < .001 as compared to control

The effect of the combination of the EGFR‐TKI erlotinib and the cMET‐TKI crizotinib was determined on different cell lines (Figure 2a). The EBC‐1 (CI, 0.66) and HCC827 (CI, 0.74) cells showed slight synergy, whereas the H1975 cell line was additive for this combination (CI, 0.93). The HCC827GR5 cell line showed very strong synergy with a CI of 0.03. To elucidate the mechanism of interaction, further studies focused on the HCC827 cell lines.

### 3.2 Effect on protein phosphorylation of EGFR and cMET

The influence of erlotinib and crizotinib on the phosphorylation of their respective targets EGFR and cMET was further evaluated using western blot analysis. In the HCC827, erlotinib alone was sufficient to inhibit phosphorylation of EGFR, while in the HCC827GR5 cells, phosphorylation of EGFR was not completely inhibited after erlotinib monotherapy (Figure 2b). However, the combination of erlotinib with crizotinib resulted in complete inhibition of EGFR‐phosphorylation. Crizotinib monotherapy of both cell lines led to complete inhibition of cMET‐phosphorylation, which was also found after combination therapy. Of note, in addition to blocking cMET phosphorylation, treatment of HCC827 and HCC827GR5 cells with crizotinib resulted in a decrease in phosphorylation of EGFR. Moreover, the treatment of both cell lines with erlotinib also resulted in a marked reduction in cMET phosphorylation. These results are in agreement with several previous studies which suggest that cMET associates with EGFR either directly, or via adapter molecules, and this association between receptors might also enable TGFα or EGF to phosphorylate cMET through EGFR. Interestingly this crosstalk might have important implications for tumorigenesis, as well as for motogenic signaling and response to therapies (Guo et al., 2008; Jo et al., 2000; Stolz & Michalopoulos, 1997; Van Der Steen et al., 2018).

### 3.3 Cell cycle and cell death analysis

The effect of the drugs on cell cycle distribution was studied at equimolar concentrations in the two cell lines, showing similar effects. Erlotinib induced a G1 arrest (Figure 2c) from 29% to 65% in the parental cell line and from 32% to 58% in the GR5 clone, respectively. Crizotinib, on the other hand, caused a G2 arrest from 21% to 57% in the HCC827 and from 26% to 71% in the HCC827GR5. The G2/M arrest was diminished in the parental cell line as compared to the control, including 39% of the cells in the parental and 49% in the HCC827GR5. Cell death was investigated as the sub‐G1 phase (Figure 2d). In the HCC827 cells erlotinib induced 20% cell death, but only 2.6% in the HCC827GR5. Despite the lack of effect of erlotinib monotherapy on the parental cell line, the combination of erlotinib and crizotinib led to approximately the same amount of cell death in both cell lines (16% in the parental and 12.5% in the HCC827GR5 cells).

### 3.4 Cell migration

For this purpose, the effect of this combination therapy on cell migration was investigated by the wound‐healing assay (Figure 2a). In the parental cell line (Figure 2b), 69 ± 10% of the scratch area was closed when treated with 0.1% DMSO (control). After crizotinib treatment 76 ± 8% of the scratch was closed, while erlotinib induced a significant reduction of migration, with 32 ± 6% of the scratch closed. Similarly, the combination of both drugs resulted in only 32 ± 5% gap closure. In the HCC827GR5 clone (Figure 2c) the control showed 80 ± 3% gap closure. Crizotinib or erlotinib treatment resulted in comparable closure with 67 ± 3% and 69 ± 2%, respectively, whereas the combination resulted in a significant reduction of migration, with 30 ± 5% closure.

### 3.5 Spheroid assay

**Figure 3:**
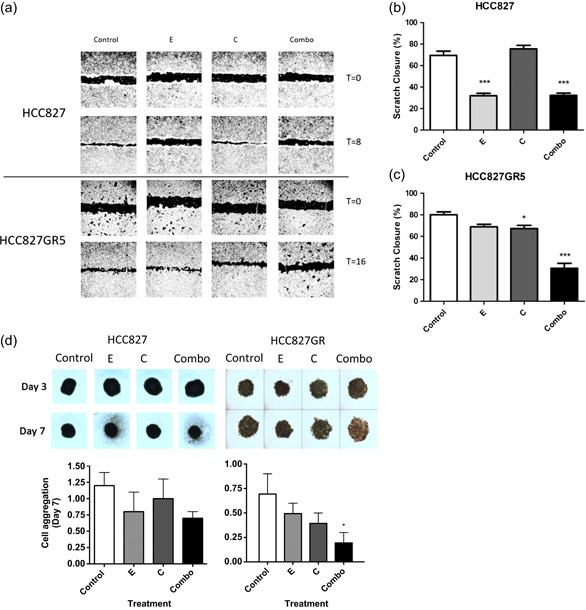
Effect of the erlotinib‐crizotinib combination on cell migration and spheroids. (a) Representative images of migration assay after 0 and 8 hr for HCC827 and 0 and 16 hr for HCC827GR5. (b) Statistical evaluation of results of the wound‐healing assay on the HCC827 cell line 8 hr after scratch induction and treatment. The percentages of scratch closure for control, erlotinib, crizotinib or erlotinib + crizotinib treated cells were compared with a one‐way analysis of variance (ANOVA) in GraphPad Prism. (c) Statistical evaluation of the results of the wound‐healing assay on the HCC827GR5 cell line 16 hr after scratch induction and treatment. The percentages of scratch closure for control, erlotinib, crizotinib or erlotinib + crizotinib treated cells were compared with a one‐way ANOVA in GraphPad Prism. (d) Effect of treatment on 3D growth. Bars represent mean ± standard error of mean after 7 days of treatment of three separate tests. Day‐3 represents spheroids immediately before the start of treatment, and Day‐7 after 96 hr of treatment. Control: 0.1% dimethyl sulfoxide, E: 10 µM erlotinib, C: 5 µM crizotinib, Combo: 10 µM erlotinib + 5 µM crizotinib. *p < .05 as compared to control, ***p < .001 as compared to control

Since previous studies illustrated that 3D cultures are generally more resistant and “closer” to solid tumors than two‐dimensional monolayer cell cultures, we assessed the effect of the different treatments in spheroids. The analysis of spheroids showed that untreated spheres appeared more compact and dense; while treated spheroids appear looser, especially at the outer layers, indicating progressive deterioration of cell‐cell interactions. This cell‐cell aggregation was measured by quantifying the amount of light passing through the spheroids, showing that erlotinib and crizotinib‐treated spheres had slightly reduced cell aggregation levels compared to untreated controls. However, the HCC827 and HCC827GR spheroids treated with the combination of erlotinib and crizotinib showed a 1.2 and 3.5‐fold reduction, respectively, further supporting the synergistic interaction in the HCC827GR cells, as reported in Figure 3.

### 3.6 Downstream signaling

**Figure 4:**
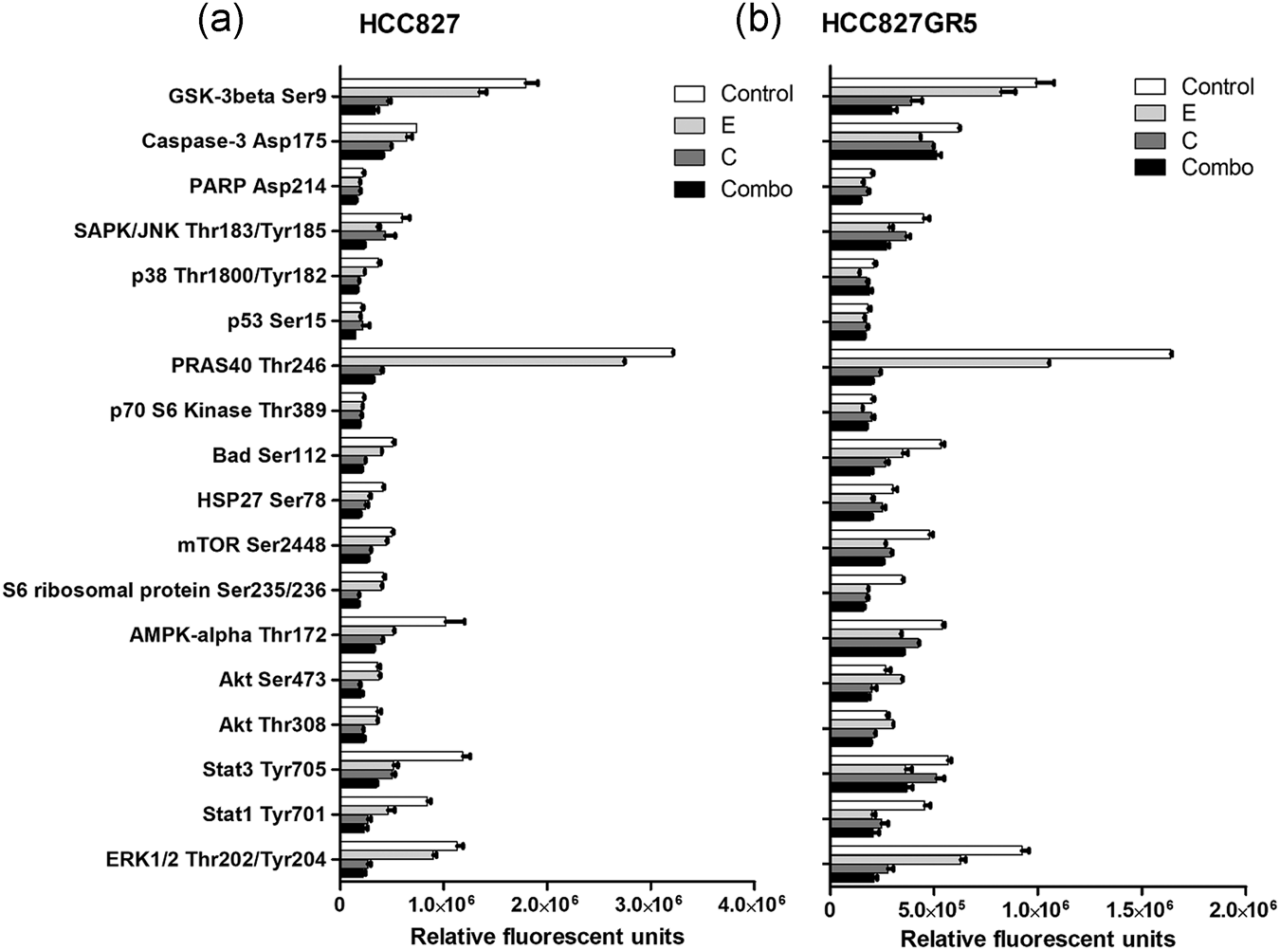
Effect of treatment on protein phosphorylation or protein cleavage. Cells were treated with 0.1% dimethyl sulfoxide (DMSO) as control, 10 µM erlotinib, 5 µM crizotinib, or a combination of both for 24 hr. Phosphorylation/protein cleavage was assessed by Pathscan assay. Relative fluorescent units were used and quantified with the Odyssey imaging system. (a) Pathscan assay HCC827; (b) Pathscan assay HCC827GR5. Control: 0.1% DMSO, E: 10 µM erlotinib, C: 5 µM crizotinib, Combo: 10 µM erlotinib + 5 µM crizotinib

A sandwich ELISA was used to determine the effect of the treatments on downstream signaling (Figure 4a,b). Differences between the parental (Figure 4a) and GR5 (Figure 4b) cell line were in general very small. Overall, changes in phosphorylation in the HCC827 are mirrored in the HCC827GR5 cell line after the different treatments. Treatment with crizotinib alone or in combination with erlotinib led to a diminished phosphorylation of ERK1/2 (30%), PRAS40 (15%), and GSKβ (30% and 38% in HCC827 and HCC827GR5, respectively) in both cell lines as compared to the untreated control, whereas phosphorylation was generally less decreased after erlotinib monotherapy. However, differences in phosphorylation levels between the HCC827 and the HCC827GR5 cell lines did not seem sufficient to explain the synergy in the HCC827GR5 cell line after combination therapy. Also, the phosphorylation in the crizotinib treated cells and in the cells treated with the combination therapy did not show statistically significant differences.

### 3.7 Peptide tyrosine kinase activity array

The kinase activity of 144 tyrosine kinases after treatment was determined using a peptide substrate array. Basal activity (Figure 4c) in the HCC827GR5 cell line is higher as compared to activity in the HCC827 parental cell line, which was set to 100%. Increased activity around 600% representing the PDGFRβ, EGFR, FGFR1, LAT, ERK5 (MK07), RON and VGFR1 was noticed. However, in the HCC827GR5 cells (Figure S1), treatment with crizotinib or with the combination showed the same level of inhibition, whereas erlotinib had minor effects on the different kinases. In the HCC827 parental cells (Figure S1), the combination treatment resulted in a slightly stronger inhibition of most kinases, whereas erlotinib or crizotinib monotherapy resulted in the same inhibition strength. Peaks in the curves, representing lower inhibition, were similar in both cell lines and occurred in the cases of FRK, paxillin, PLCγ1, cortactin (SRC8_CHICK) and VGFR2. However, since this pattern was found in both the parental and resistant cell line, this did not explain the synergy in the HCC827GR5. Generally, HCC827 cells showed a 20% inhibition of kinase activity after combination treatment as compared to monotherapy. Of note, the kinase activity of FRK, cortactin, and paxilin increased after erlotinib or crizotinib monotherapy, whereas combination therapy led to an activity comparable to the untreated cells. All three kinases are downstream of receptor tyrosine signaling. In the HCC827GR5 cell line, the curves of crizotinib monotherapy and combination treatment showed the same value for all kinases, whereas erlotinib led to a slight decrease (about 10–20%) in activity on average. Treatment with crizotinib, whether or not in combination with erlotinib, led to an activation of EFS (x3), FRK (x3.5), paxillin (x2), PLCγ1 (2 × ), RET (2 × ) and cortactin (x5). EFS, FRK, paxillin, PLCγ1, and cortactin are downstream effectors of tyrosine kinase receptors, while RET was the only tyrosine kinase receptor that was more activated in this analysis.

### 3.8 Intracellular drug concentration

**Figure 5:**
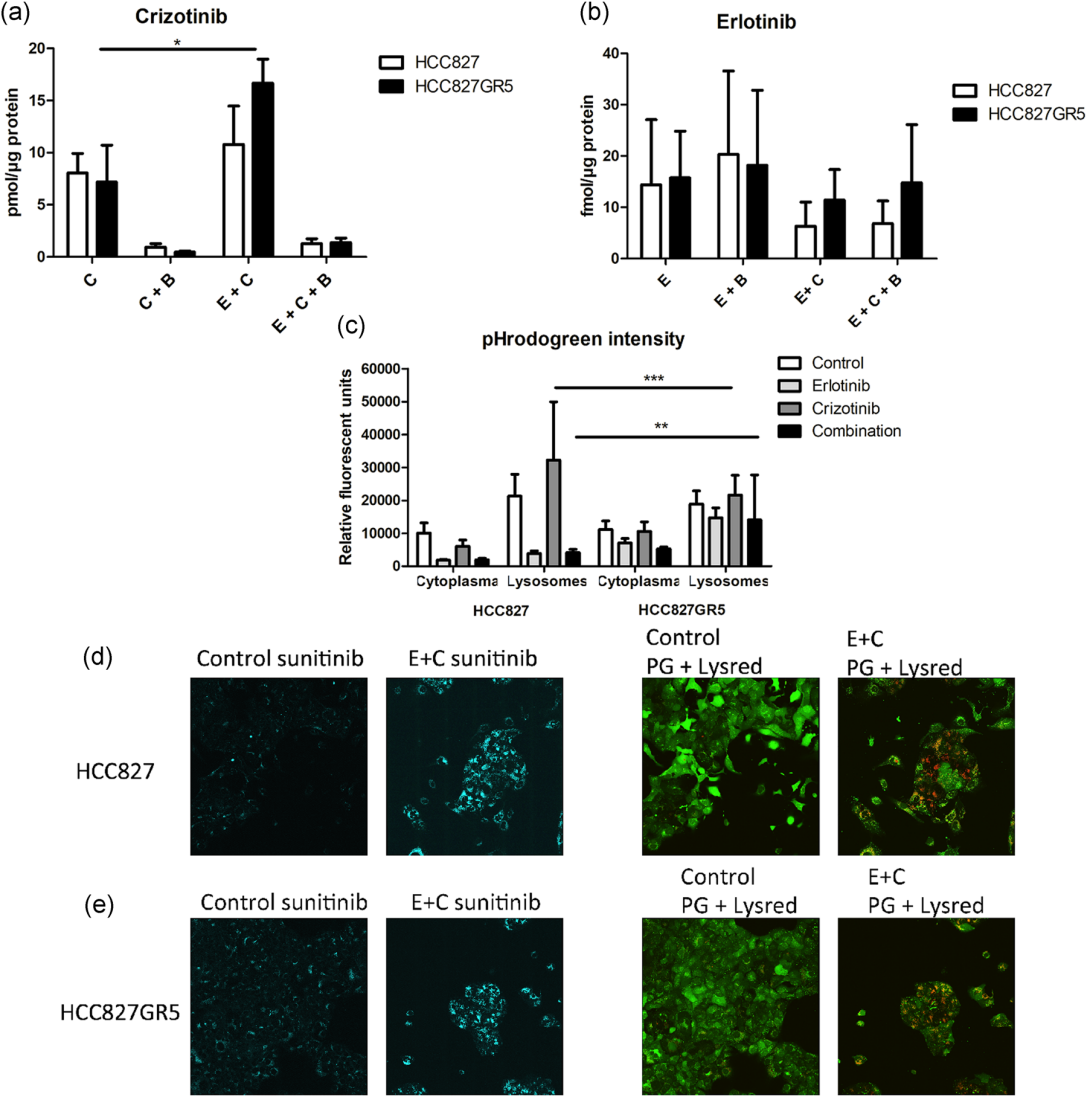
Effect of erlotinib on the intracellular and lysosomal accumulation of crizotinib and on the role of pH. Cells were treated with 0.1% dimethyl sulfoxide as control, 10 µM erlotinib, 5 µM crizotinib or their combination for 24 hr. Bafilomycin (50 nM) was used to perturb the lysosomal function. (a) Effect of erlotinib on the intracellular crizotinib concentration in pmol/µg protein. (b) Intracellular erlotinib concentration in fmol/µg protein. Bars represent mean ± standard error of mean of three separate tests. (c) Quantification of pHrodogreen intensity in relative fluorescent units with FIJI from d, e. All the *p*‐values are summarized in Table S1. (d, e) Intracellular effect of drugs on HCC827 and HCC827GR5, respectively, cells were stained for 1 hr with 5 µM sunitinib and 0.5 µM Lysotracker Red, and for 30 min with pHrodoGreen. Each sample was divided in multiple focus planes (z‐stack). Z‐stacks were imaged using a Leica TCS SP8 STED 3× microscope. Image panels of d from left to right: sunitinib staining of HCC827 control cells; sunitinib staining of HCC827 treated with 10 µM erlotinib and 5 µM crizotinib; Phrodogreen and Lysotracker red staining of HCC827 control cells; Phrodogreen and Lysotracker red staining of HCC827 treated with 10 µM erlotinib and 5 µM crizotinib. Image panels of e from left to right: sunitinib staining of HCC827GR5 control cells; sunitinib staining of HCC827GR5 treated with 10 µM erlotinib and 5 µM crizotinib; Phrodogreen and Lysotracker red staining of HCC827GR5 control cells; Phrodogreen and Lysotracker red staining of HCC827GR5 treated with 10 µM erlotinib and 5 µM crizotinib. Control: 0.1% DMSO, E: 10 µM erlotinib, C: 5 µM crizotinib, Combo: 10 µM erlotinib + 5 µM crizotinib, B: 50 nM Bafilomycin A1. *p < .05 as compared to control, **p < .01; ***p < .001 as compared to control

Since previous studies on TKIs have shown that the effect of drugs on cell cycle and cell death is dependent on their uptake, we determined the intracellular concentrations of crizotinib (Figure 5a) and erlotinib (Figure 5b) in both cell lines. Concentrations of crizotinib after monotherapy were similar in the HCC827 and HCC827GR5 cells (8 and 7 pmol/µg protein, respectively). However, crizotinib levels in the HCC827GR5 cells increased significantly after co‐treatment with erlotinib (*p* < .05), whereas this increase was only up to 11 pmol/µg protein in the HCC827 cells. A statistically significantly higher concentration might not be biologically relevant. However, in our experiments, the treatment with erlotinib increased the concentration of crizotinib more than two‐fold in the HCC827GR5 (lysosomes and cytoplasma: 17 pmol/μg protein) and we hypothesize that the latter will enable a better inhibition of cMET in the HCC827GR5 cells in the cytosol.

To further investigate the intracellular concentration, we measured crizotinib trapping in the lysosomes. A concentration of 50 nM of bafilomycin A1, which was previously reported to cause lysosomal dysfunction (Bowman, Siebers, & Altendorf, 1988; Yoshimori, Yamamoto, Moriyama, Futai, & Tashiro, 1991), and prevent accumulation of sunitinib and other tyrosine kinase inhibitors in the lysosomes (Gotink et al., 2011) led to a strong decrease to concentrations around 1 pmol/µg protein in the parental and the gefitinib resistant variant. In the combination of erlotinib and crizotinib, bafilomycin also decreased crizotinib accumulation, but the total free concentration of crizotinib was more than two‐fold higher compared to crizotinib alone, possibly allowing more pronounced inhibition of the cMET pathway.

Conversely, erlotinib concentrations were similar in both cell lines and were not affected by bafilomycin, showing no lysosomal involvement.

The difference in crizotinib uptake between both cell lines could be due to a difference in expression of ATP binding cassette transporters, of which PGP and MRP1 are the most common ones. However, the Western Blot analysis (Figure 2b) showed that PGP was not expressed in both cell lines. A difference in expression of MRP1 was found, showing a slightly higher expression of MRP1 in the HCC827GR5 cells. However, this slight difference is unlikely to cause a difference in intracellular crizotinib levels. Indeed our experiments with the MK571 inhibitor of MRP1 did not impact drug sensitivity (Figure S2).

### 3.9 Intracellular effect of drugs

Live cell microscopy was used to determine the intracellular pH of the cytoplasm and lysosomes of both the HCC827parental (Figure 5c,d) and HCC827GR5 (Figure 5c–e) cell lines. Representative images showed overlap between lysotracker red and bright spots in the pHrodoGreen channel. There was also an overlap between sunitinib (blue channel) and lysotracker red, showing sunitinib accumulation in the lysosomes in both cell lines. The overlay between lysotracker red and brightfield images showed that the lysotracker red signal was mainly located around the nucleus. Treatment with erlotinib did not result in a visual change, whilst crizotinib and the combination led to an increased number of lysosomes and increased sunitinib signal.

The intensity of pHrodoGreen was quantified and corrected for the area of the cells and the number of z‐planes in the respective stacks (Figure 5c). The corresponding *p*‐values are listed in Table S1. The higher the intensity of the pHrodoGreen, the lower the pH. The basal pH of cytoplasm and lysosomes was not different between the parental and GR5 cell lines in the control samples. In the parental cell line, the intensity of pHrodoGreen in both the cytoplasm and lysosomes was very low, indicating an alkaline pH. This effect was probably due to cell death in response to erlotinib. Crizotinib caused a rise in pH in the cytoplasm and a large decrease in pH within the lysosomes. However, after combination therapy, the effect of erlotinib was dominant. In the HCC827GR5 cell line, erlotinib treatment resulted in an increased pH in both the cytoplasm and lysosomes. Crizotinib, on the other hand, did not alter the pH much in both compartments. In combination, the effect of erlotinib was dominant also in this cell line.

## 4 DISCUSSION

The present study supports a potential role for both genetic properties and lysosomal function in the synergistic interaction of erlotinib and crizotinib in NSCLC cells.

This novel finding was obtained by evaluating the HCC827 cell line, which harbors an EGFR exon 19 deletions, and the HCC827GR5 cells, which also harbors the cMET amplification as a resistance mechanism to gefitinib. Combined treatment with the EGFR‐TKI erlotinib and the cMET‐TKI crizotinib resulted in a very strong synergy in the HCC827GR5 cells. In contrast, no synergy was observed in the cMET amplified EBC‐1 or the EGFR mutated H1975 (L858R +  T790M) cell lines.

The signaling pathways of cMET and EGFR are strongly intertwined (Breindel et al., 2013; Guo et al., 2008) which could result in synergism between erlotinib and crizotinib. However, the EBC‐1 cell line which is wild‐type for EGFR and harbors a cMET amplification only showed an additive effect of the combination. An activating EGFR mutation seems therefore essential for synergism. Altogether these data prompted us to focus on the HCC827GR5 cells and compare all experiments to the parental HCC827 cell line.

The synergy between erlotinib and crizotinib was also reflected in the wound‐healing assay, where only the combination was able to inhibit cell migration in the HCC827GR5 cell line. Activation of cMET has been shown to stimulate cell migration (Birchmeier, Birchmeier, Gherardi, & Vande Woude, 2003; Bladt, Riethmacher, Isenmann, Aguzzi, & Birchmeier, 1995) and the same holds true for EGFR (Carcereny et al., 2015; Ramis, Thomàs‐Moyà, de Mattos, Rodríguez, & Villalonga, 2012). However, it is remarkable that the cell line harboring both aberrations migrates twice as slow as compared to the parental cell line, which is reflected in the time it takes for the control wound to close (16 vs. 8 hr).

Analysis of the sub‐G1 fraction is also in line with the found synergy in the HCC827GR5 cell line, whereas in the parental cell line the effect of erlotinib was slightly stronger than the combination. Further, the cell cycle analysis showed a G1 arrest after erlotinib (Giovannetti et al., 2008; Huether, Höpfner, Sutter, Schuppan, & Scherübl, 2005) and a G2/M arrest after crizotinib (Megiorni et al., 2015), which is in accordance with the literature. However, after the combination, the effect of crizotinib was dominant but weaker than after the monotherapy.

Remarkably, this synergy was further reflected when comparing 3D cultures of both cell lines. Through the analysis of cell aggregation of the parental HCC827 and the HCC827GR lines, which had a different growth pattern in 3D, with a recently validated method, we indeed found that the combination significantly reduced cellular aggregation of the spheroids in the HCC827GR, while only a slight reduction was detected in the HCC827 cells.

To determine whether the synergy is due to the combined inhibition of cMET and EGFR downstream signaling or if other mechanisms play an important role, we compared downstream phosphorylation of the parental cell line and the HCC827GR5 clone. Overall, both cell lines reacted very similarly to the different treatment conditions, not giving a satisfactory explanation for the synergy in the HCC827GR5 cells. The highest differences in basal activity, around 600%, were observed for several tyrosine receptor kinases. However, in the HCC827 parental cell line, the activity of these kinases was below the baseline, whereas in the HCC827GR5 activity of these kinases was just above the baseline. This makes the comparison between these values very difficult. Moreover, in both the HCC827 and HCC827GR5 cells, the activities of kinases after treatment with crizotinib monotherapy or with the combination were approximately the same.

We next focused on the cellular pharmacokinetics of the drugs. Crizotinib and gefitinib are both known as hydrophobic weak‐base compounds (Da Silva et al., 2015). These weak bases are able to freely cross the plasma membrane and intracellular membranes. The compounds might also accumulate in the lysosomes, and this accumulation is driven by pH partitioning between the cytoplasm and the lysosomes (Zhitomirsky & Assaraf, 2015b). The low pH in the lysosomes causes protonation of several weak‐base TKIs, thus preventing the membrane‐crossing of these bases and leading to accumulation in the lysosomes (Gotink et al., 2011). This results in lysosomal stress and activation of the coordinated lysosomal expression and regulation (CLEAR‐pathway) by the TFEB transcription factor. This accumulation may also trigger the activation of lysosomal exocytosis, whereby lysosomes travel over the microtubuli, fuse with the plasma membrane and release their cargo into the extracellular environment (Zhitomirsky & Assaraf, 2015a, 2017). Erlotinib, on the other hand, is associated with the cytoskeleton (Da Silva et al., 2015).

Of note, the intracellular concentration of crizotinib varied significantly between treatment conditions in the HCC827GR5, with a significant increase in concentration after combination treatment as compared to crizotinib monotherapy. Although we can only hypothesize that this increase would have clinically relevant effects, a similar increase in the concentration of crizotinib was associated with relevant synergistic interaction in our in vivo models of pancreatic cancer (Avan et al., 2013).

By the addition of bafilomycin, the intracellular concentration of crizotinib decreased to 1 pmol/µg protein in all treatment conditions. This demonstrates that crizotinib is sequestered in the lysosomes (Da Silva et al., 2015) and that the combination possibly influences the process of lysosomal sequestration.

This effect might be caused by an alteration of the pH partitioning between the cytoplasm and the lysosomes (Zhitomirsky & Assaraf, 2015b), as has been shown in the MCF‐7 cell line (Zhitomirsky & Assaraf, 2015b). A decrease in the pH difference between both compartments might result in decreased sequestration of crizotinib in the lysosomes. The addition of erlotinib might normalize the pH, leading to renewed sequestration of crizotinib. However, live‐cell microscopy with the pH‐sensitive probe pHrodoGreen showed no difference in basal pH level between the parental and HCC827GR5 cell lines. Moreover, in both cell lines sunitinib accumulated in the lysosomes, showing that lysosomal sequestration is ongoing in both cell lines (Gotink et al., 2011). However, the acidification of the lysosomes after crizotinib treatment in the HCC827 parental cells could explain the higher intracellular concentration of crizotinib in the cells, whereas the pH assessment after the combination is complicated due to the large increase in the area of the lysotracker red signal because of ongoing apoptosis. These combined results show that there is no permanent change in pH in the HCC827GR5 cell line and that lysosomal accumulation of sunitinib, and crizotinib, still takes place.

Another explanation of our results might be the influence of erlotinib on autophagy. Several studies have shown the influence of erlotinib, and EGFR‐TKIs in general, on the induction of autophagy (Han et al., 2011; Li, Lam, Mak, Zheng, & Ho, 2013; Nihira et al., 2014). This induction of autophagy was observed both in EGFR mutated and EGFR‐independent cells (Han et al., 2011). In the process of autophagy (White, 2012), cell compartments destined for breakdown are enveloped by a membrane and fuse with the lysosomes. Next, the proteinases in the lysosomes break down the cell compartment. It has been shown that upon the accumulation of lysosomotropic compounds, the pH of the lysosomes is increased (Lu, Jessen, Strock, & Will, 2012). In turn, this increase in lysosomal pH can prevent the optimal functioning of lysosomal proteases, ultimately leading to impairment of autophagy. Moreover, lysosomal accumulation by itself is enough to activate autophagy. The transcription factor TFEB, which is activated upon lysosomal accumulation, translocates to the nucleus and activates the expression of genes involved in lysosomal biogenesis, autophagy, and endocytosis (Settembre et al., 2011). The combination of erlotinib with crizotinib treatment might thus lead to enhancement of the impaired autophagy process, resulting in increased cell death. Of note, previous studies showed that enhancement of autophagy is associated with an increase in p‐AMPK (Thr172) and a decrease in p‐mTOR (Ser2448; Li et al., 2013). However, our data on phosphorylation show no increase in p‐AMPK in the HCC827GR5 cell line after combination therapy as compared to the monotherapies, nor a large decrease in p‐mTOR, contradicting this hypothesis.

A third explanation might be that the association of erlotinib with the cytoskeleton interferes with lysosomal exocytosis over the microtubule trackers (Zhitomirsky & Assaraf, 2017), leading to an increase in intracellular crizotinib after combination therapy as compared to monotherapy. However, there are no studies determining in detail the interaction of erlotinib with the cytoskeleton and its influence on the microtubule racks.

## 5 CONCLUSION

Both genetic characteristics and lysosomal function seem to contribute to the synergistic interaction between crizotinib and erlotinib in the HCC827GR5 cell line, and more research is warranted to determine the exact mechanisms linking these cellular properties. Moreover, future studies in clinical specimens would be essential to evaluate whether genetic background and lysosomal sequestration could guide tailored therapeutic interventions with the crizotinib–erlotinib combination in NSCLC patients.

## Supporting information

Supporting information

Supporting information

Supporting information

Supporting information

## Data Availability

The data that support the findings of this study are available on request from the corresponding author.
