# Supplementary material for: Crizotinib sensitizes the erlotinib resistant HCC827GR5 cell line by influencing lysosomal function
Source: J Cell Physiol. 2020 Jan 20;235(11):8085–97. doi: 10.1002/jcp.29463 (PMC7540474; doi:10.1002/jcp.29463)
Supplement: Supplementary file 1 — Supporting information [file JCP-235-8085-s001.docx]

| Supplementary table 1: p-values pHrodoGreen intensity | | | |
| --- | --- | --- | --- |
| Condition 1 | Condition 2 | cell organelle | p-value |
| HCC827 control | HCC827 E | cytoplasm | p<0.01 |
|  |  | lysosomes | p<0.001 |
|  | HCC827 C | cytoplasm | ns |
|  |  | lysosomes | p<0.001 |
|  | HCC827 E+C | cytoplasm | p<0.01 |
|  |  | lysosomes | p<0.001 |
|  | HCC827GR5 control | cytoplasm | ns |
|  |  | lysosomes | ns |
|  | HCC827GR5 E | cytoplasm | ns |
|  |  | lysosomes | p<0.05 |
|  | HCC827GR5 C | cytoplasm | ns |
|  |  | lysosomes | ns |
|  | HCC827GR5 E+C | cytoplasm | ns |
|  |  | lysosomes | p<0.05 |
| HCC827 E | HCC827 C | cytoplasm | ns |
|  |  | lysosomes | p<0.001 |
|  | HCC827 E+C | cytoplasm | ns |
|  |  | lysosomes | ns |
|  | HCC827GR5 control | cytoplasm | p<0.01 |
|  |  | lysosomes | p<0.001 |
|  | HCC827GR5 E | cytoplasm | ns |
|  |  | lysosomes | p<0.001 |
|  | HCC827GR5 C | cytoplasm | p<0.01 |
|  |  | lysosomes | p<0.001 |
|  | HCC827GR5 E+C | cytoplasm | ns |
|  |  | lysosomes | p<0.001 |
| HCC827 C | HCC827 E+C | cytoplasm | ns |
|  |  | lysosomes | p<0.001 |
|  | HCC827GR5 control | cytoplasm | ns |
|  |  | lysosomes | p<0.001 |
|  | HCC827GR5 E | cytoplasm | ns |
|  |  | lysosomes | p<0.001 |
|  | HCC827GR5 C | cytoplasm | ns |
|  |  | lysosomes | p<0.001 |
|  | HCC827GR5 E+C | cytoplasm | ns |
|  |  | lysosomes | p<0.001 |
| HCC827 E+C | HCC827GR5 control | cytoplasm | p<0.01 |
|  |  | lysosomes | p<0.001 |
|  | HCC827GR5 E | cytoplasm | ns |
|  |  | lysosomes | p<0.001 |
|  | HCC827GR5 C | cytoplasm | p<0.01 |
|  |  | lysosomes | p<0.001 |
|  | HCC827GR5 E+C | cytoplasm | ns |
|  |  | lysosomes | p<0.001 |
| HCC827GR5 control | HCC827GR5 E | cytoplasm | ns |
|  |  | lysosomes | ns |
|  | HCC827GR5 C | cytoplasm | ns |
|  |  | lysosomes | ns |
|  | HCC827GR5 E+C | cytoplasm | ns |
|  |  | lysosomes | ns |
| HCC827GR5 E | HCC827GR5 C | cytoplasm | ns |
|  |  | lysosomes | p<0.05 |
|  | HCC827GR5 E+C | cytoplasm | ns |
|  |  | lysosomes | ns |
| HCC827GR5 C | HCC827GR5 E+C | cytoplasm | ns |
|  |  | lysosomes | ns |

Cells were treated with erlotinib (E), crizotinib (C) or the combination (E+C) for 24 h and stained with pHrodoGreen, sunitinib and lysotracker Red. Images were analyzed with FIJI as described in Figure 1. Statistical analysis was performed with Graphpad prism v5. Corresponding p-values are depicted in this table. ns: not significant.
