# Supplementary material for: Crizotinib sensitizes the erlotinib resistant HCC827GR5 cell line by influencing lysosomal function
Source: J Cell Physiol. 2020 Jan 20;235(11):8085–97. doi: 10.1002/jcp.29463 (PMC7540474; doi:10.1002/jcp.29463)
Supplement: Supplementary file 2 — Supporting information [file JCP-235-8085-s002.docx]

**Legends to Supplementary Figures:**

**Figure 1**: Effect of treatment on protein phosphorylation or protein cleavage. Cells were treated with 0.1% DMSO as control, 10 µM erlotinib, 5 µM crizotinib or a combination of both for 24 h. Phosphorylation/protein cleavage was assessed by Pathscan assay. a) Relative kinase activity (control treatment) of HCC827GR5 as compared to HCC827 (set at 100%); Effect of erlotinib, crizotinib and the combination on kinase activity of HCC827 (b) and HCC827GR5 (c). Controls (untreated) were set at 100%.

**Figure 2**: Evaluation of cell growth inhibition of crizotinib alone and combined with the MRP1 inhibitor MK571 (20 μM) on HCC827 cells. Cell growth inhibition was calculated as the percentage OD of drug-treated cells versus that of vehicle-treated cells (negative control) (corrected for OD before drug addition, day-0). Finally, the half maximal-inhibitory concentration (IC50) was calculated with GraphPad Prism 7 (Intuitive Software for Science, San Diego, CA, USA). The IC50 was calculated by non-linear least-squares curve fitting. Values represent mean ± SEM of three separate tests.
