# Supplementary figures and images for: Crizotinib sensitizes the erlotinib resistant HCC827GR5 cell line by influencing lysosomal function
Source: J Cell Physiol. 2020 Jan 20;235(11):8085–97. doi: 10.1002/jcp.29463 (PMC7540474; doi:10.1002/jcp.29463)

Supplementary Figure 1

A

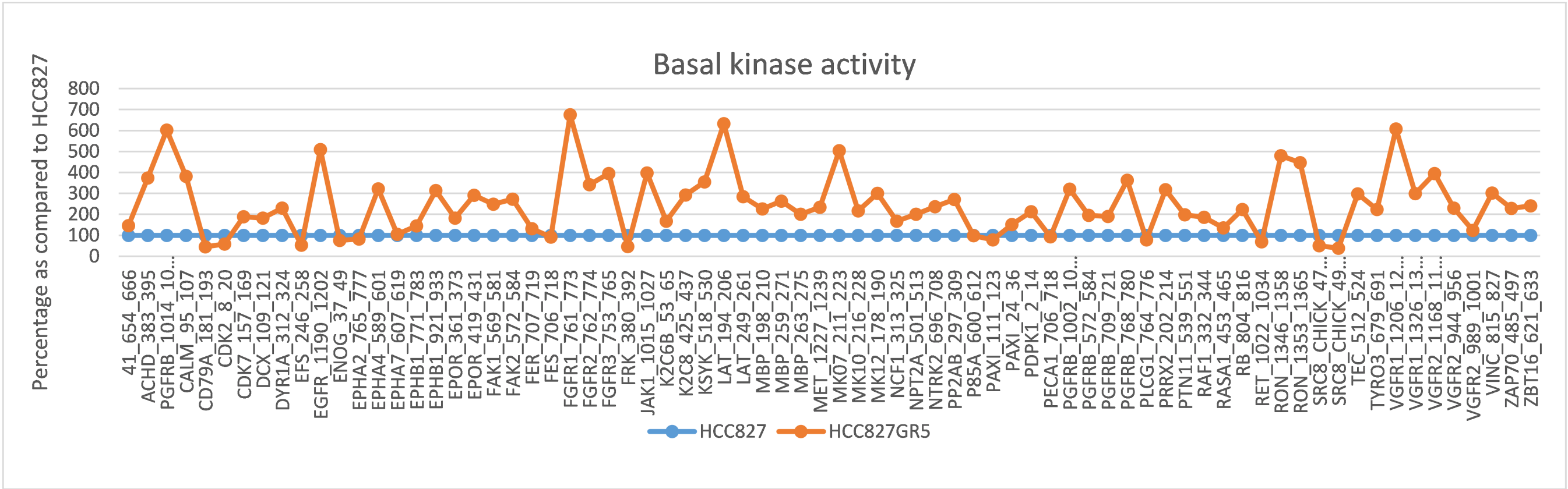

B

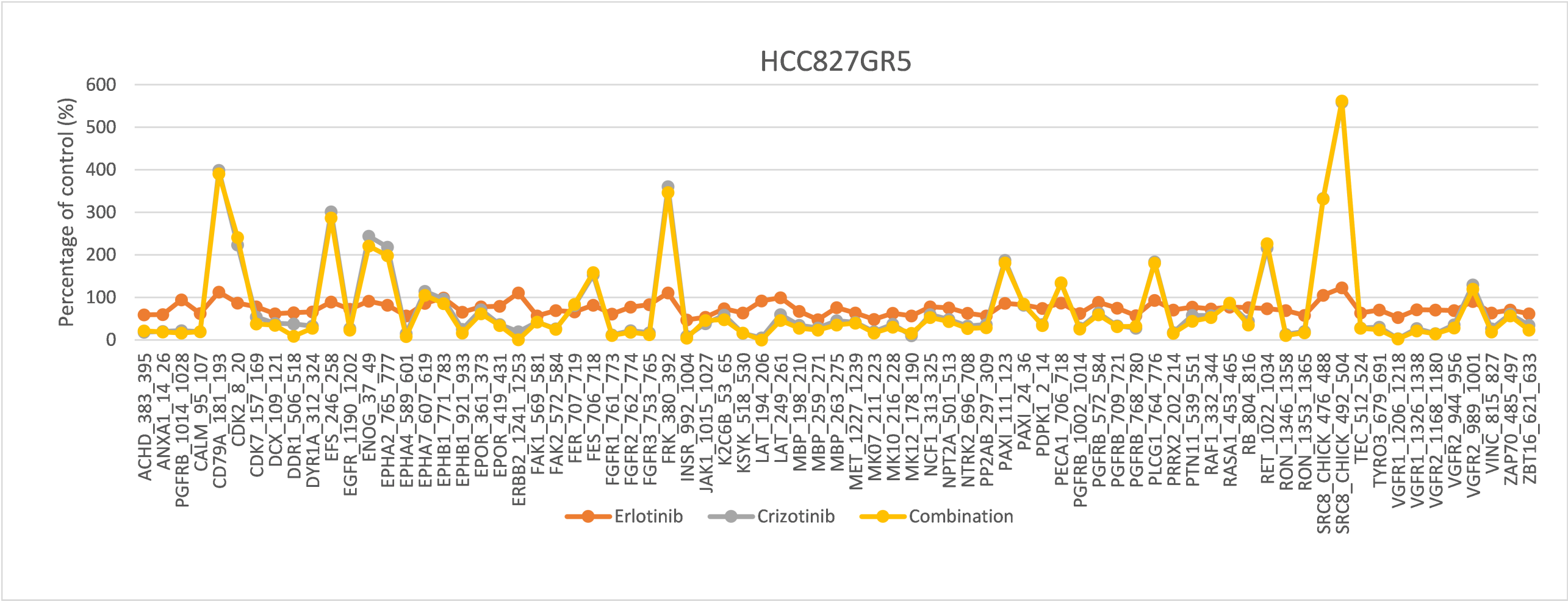

C

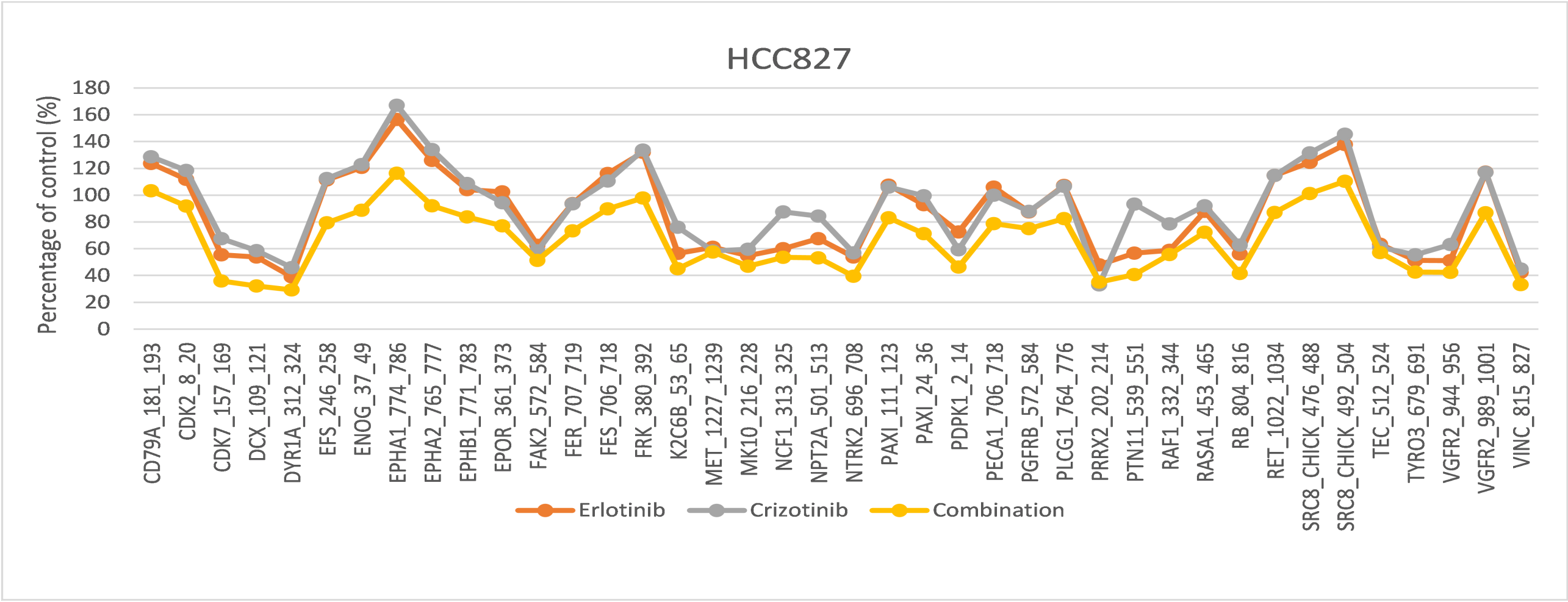

Supplement: Supplementary file 3 — Supporting information [file JCP-235-8085-s003.pdf]

Supplementary Figure 2

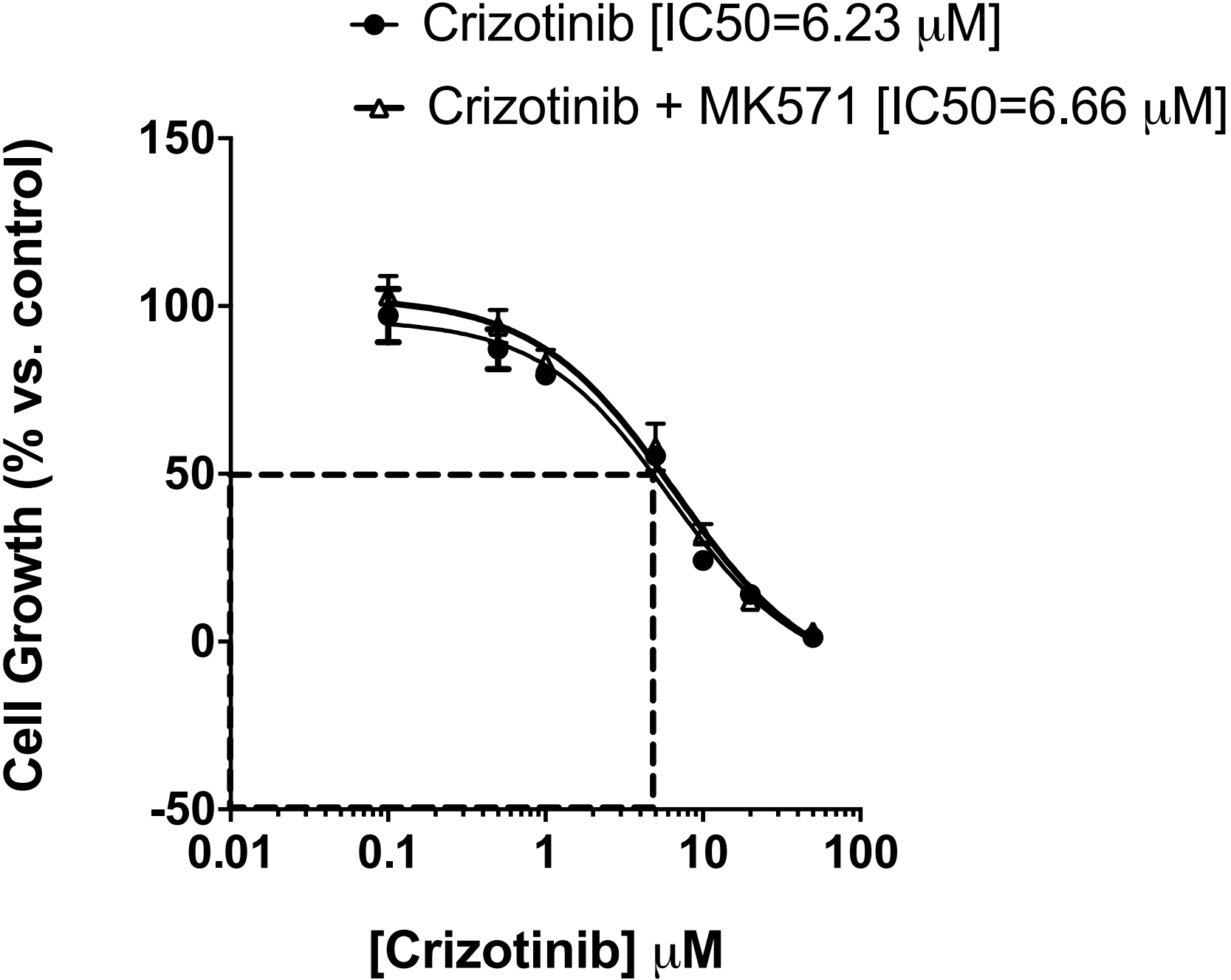

Supplement: Supplementary file 4 — Supporting information [file JCP-235-8085-s004.pdf]
